# Forest tree–pathogen interactions under climate change

**DOI:** 10.1042/EBC20250054

**Published:** 2026-07-30

**Authors:** Fred O. Asiegbu, Wenjing Meng, Wenzi Ren, Guiyang Yang, Abiodun Azeez, Yilin Li, Ziwen Gao, Zilan Wen, Kai Wang, Victor Chano

**Affiliations:** 1 Faculty of Agriculture and Forestry, Department of Forest Sciences, University of Helsinki, Helsinki, Finland; 2 Walnut Engineering Technology Research Center of Shaanxi Province, College of Forestry, Northwest Agriculture & Forestry University, Yangling, China; 3 College of Forestry, Fujian Agriculture and Forestry University, Fuzhou, China; 4 Department of Forest Genetics and Forest Tree Breeding, University of Gottingen, Germany

**Keywords:** Biotic-abiotic factors, Climate change, Forest Pathology, mycology, Resistant Biology, Tree-microbe interactions

## Abstract

Forest trees are ecologically and economically vital, contributing to carbon sequestration, biodiversity conservation, pollution mitigation, and renewable bioenergy. However, forests worldwide are increasingly threatened by interacting biotic and abiotic stressors, including pathogens, insect pests, drought, heat, and extreme weather events driven by climate change. Drought and heat stress trigger complex physiological and metabolic responses in trees that can reshape their resistance to pathogens. While moderate stress may activate protective mechanisms, severe stress can cause cellular damage, dehydration, and reactive oxygen species accumulation, weakening defense capacity. Emerging evidence highlights additional layers of regulation, including epigenetic mechanisms and the role of beneficial microbiomes in enhancing tree resilience under combined environmental and pathogen pressures. Breeding and genetic improvement are also essential for strengthening adaptation and resistance to emerging diseases. Although advances in forest genetics, long-term field studies, and tree genomics are improving our predictive capacity, major knowledge gaps remain. Addressing how forest trees respond to pathogens under climate change will require multidisciplinary approaches integrating molecular biology, multiomics, big data analytics, remote sensing, ecology, and climate modelling.

## Introduction- forests in a changing climate: importance and emerging challenges

**Figure 1:**
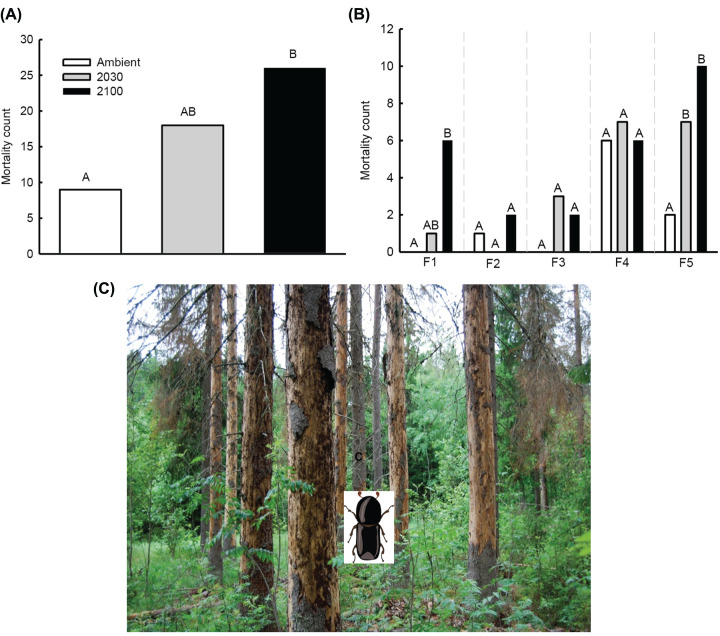
Impact of projected climate change on Norway spruce resistance to *Endoconidiophora polonica* infection Seedling mortality at the end of the experiment adapted from Linnakoski et al. [6]: (**A**) combined across all fungal strains of *Endoconidiophora polonica* and (**B**) by individual fungal strains. Strain effects were identified using generalized linear models (F4 and F5) and chi-squared tests (F1, F2, and F3). Different letters above the bars indicate statistically significant differences among climate change scenarios (*P*<0.05). (**C**) Bark beetle infested tree (picture by Riikka Linnakoski). The present work is licensed under a CC BY 4.0 (http://creativecommons.org/licenses/by/4.0/)

Forests occupy approximately 31% of the Earth’s land surface (4.06 billion ha) and remain foundational to global ecological stability, containing nearly 80% of terrestrial plant biomass and contributing about 75% of global gross primary production [1,2]. Their disproportionate concentration in a few countries and the persistence of relatively intact primary forests highlight both their strategic conservation value and vulnerability to accelerating environmental change [3]. Beyond carbon sequestration and climate regulation [4], forests sustain biodiversity, support soil and water regulation, and underpin a growing bioeconomy through timber and renewable resources. However, their ecological and economic importance is increasingly challenged by interacting biotic and abiotic stressors [5,6]. Forest health is under growing pressure from pathogens, invasive species, insect pests, and climate-driven disturbances such as drought and heat extremes (Figure 1) [7–9]. Critically, these pressures do not operate in isolation; rather, their interactions often intensify forest decline [10,11]. Host-jumping pathogens and invasive species represent a particularly significant threat, reshaping ecosystems and causing ecological and economic disruption [12]. Outbreaks such as chestnut blight, Dutch elm disease, ash dieback, and *Xylella fastidiosa* illustrate how disease can rapidly transform forest landscapes. From a critical standpoint, these examples expose the limitations of conventional reactive disease management and suggest a need to shift toward more integrated, ecologically grounded approaches centered on tree–microbe interactions, pathogen ecology, and long-term resilience.

Climate change further complicates this challenge by altering disease dynamics through warming temperatures, elevated CO_2_, shifting precipitation patterns, and more frequent extremes [6,13]. Evidence suggests climate warming may accelerate pathogen life cycles, expand pest and pathogen ranges poleward, and intensify drought-mediated vulnerability, particularly in boreal systems where fungal pathogens and bark beetle interactions may be amplified [5,13–16]. In our view, this reinforces a critical conceptual shift: forest disease should not be treated solely as a pathology problem but as a climate-mediated ecological process. This perspective has important implications for breeding and forest management, which must prioritize adaptive capacity alongside resistance to pests and diseases. Understanding how water stress shapes disease development in key pathosystems is a scientific priority; therefore, it is essential for anticipating future forest resilience and informing sustainable management under climate uncertainty.

## Mechanisms and ecological dynamics of tree–pathogen interactions

Forest health is threatened by diverse pathogens: fungi, oomycetes, bacteria, viruses, nematodes, and phytoplasmas, whose impacts are often amplified through interactions with insect pests and abiotic stress [16,17]. While native pathogens are often moderated by co-evolved host resistance, introduced pathogens can be highly destructive due to ecological mismatch and low host preparedness [18]. Fungi and oomycetes remain the dominant drivers of disease, causing wilts, cankers, rots, and decline syndromes [19], while bacteria, phytoplasmas, viruses, and nematodes contribute to vascular dysfunction, chronic decline, and predisposition to secondary infection. A critical implication is that forest disease should be viewed less as single-pathogen outbreaks and more as stress-mediated pathosystems shaped by interacting biotic and abiotic drivers. Tree defenses against these threats rely on multilayered constitutive and induced mechanisms, including bark structure, resin chemistry, phenolics, suberization [20,21], and immune signaling pathways [22–27] (see below). Compounds such as terpenoids, stilbenes, and flavonoids contribute to broad-spectrum resistance [21], while PTI/ETI-mediated responses coordinate reactive oxygen species (ROS) signalling, hypersensitive response, and hormone-regulated defense (see below). However, a key uncertainty is how climate change modifies these defenses. Evidence suggests warming, drought, and elevated CO_2_ may disrupt carbon allocation, hormone balance, and secondary metabolism, potentially weakening resistance, but empirical tests integrating climate stress into phytopathology systems remain limited. Future studies need to move beyond static defense models to experimentally resolve how multiple climate factors alter resistance mechanisms.

These responses are further constrained by growth-defense trade-offs. Induced resistance carries carbon and nutrient costs that can reduce growth and productivity [28], and climate stress may intensify these trade-offs by altering resource availability and lignification. Critically, resistance under future climates may depend less on maximizing defense and more on maintaining adaptive balance between growth and protection, an issue still under-represented in forest pathology. At the evolutionary scale, plant–pathogen interactions reflect dynamic co-evolution shaped by gene-for-gene processes [29], but climate change and global trade are accelerating disruptions to these equilibria [30]. Emerging diseases such as *Diplodia sapinea* in Finland and Beech Leaf Disease in North America [31,32] illustrate how range shifts, ecological fitting, hybridization, and anthropogenic selection can rapidly generate novel pathosystems [33,34]. This challenges traditional frameworks centered on gradual adaptation, suggesting that evolutionary mismatch may become a defining feature of future forest disease dynamics. Strengthening predictive pathology will therefore require integrating ecology, evolution, invasion biology, and climate-driven host susceptibility into monitoring and risk models [35,36].

## Climate change drivers shaping tree–pathogen interactions: temperature, drought, and heat stress

Rising temperatures intensify forest disease by affecting both pathogen performance and host susceptibility. Temperature regulates incubation, spore germination, mycelial growth, and infection processes [37,38], while warming often enhances pathogen survival, reproduction, and epidemic cycling through shortened incubation and increased sporulation [38]. In tropical and subtropical systems, *Thielaviopsis paradoxa* shows peak aggressiveness at 27°C–33°C, particularly during hot, dry periods, contributing to severe neck-bending disease outbreaks in oil palm [39]. Its thermal optimum aligns with that of its host, supporting its prevalence in these regions. In temperate forests, warming especially mild winters and longer growing seasons has increased disease severity associated with pathogens such as *D. sapinea*, *Heterobasidion* spp., and *Ophiostoma novo-ulmi*. Heat combined with drought further weakens host defenses and increases susceptibility [40,41]. Climate-driven shifts also favor insect–pathogen complexes; simulations project major poleward expansion of bark beetles by 2081–2100, with northern Europe increasingly vulnerable to multi-species invasions [14].

Drought exerts contrasting effects across disease types, often suppressing foliar pathogens while intensifying stem and root diseases [42]. Through stomatal closure, reduced defense metabolites, fine-root loss, and carbon starvation, drought increases susceptibility, particularly to necrotrophs [43]. Together with pathogens, extreme drought is a major driver of global forest decline [44]. Increased precipitation variability (∼40%) [45] further reshapes disease dynamics: rain splash enhances dispersal, humidity may suppress stomatal immunity [46,47], and waterlogging induces hypoxia and metabolic disruption that favour oomycetes such as *Phytophthora* [48,49]. However, long-term microbiome analyses from 150-year herbarium needles showed only weak climatic correlations, despite persistent occurrence of pathogens such as *Heterobasidion parviporum* and *Lophodermium piceae* [50], suggesting unresolved complexity in long-term climate–pathogen interactions.

Rising greenhouse gases add further complexity by altering host physiology. Elevated CO_2_ often increases plant C:N ratios [51], reducing tissue quality for herbivores [52] while modifying nitrogen-dependent defenses [53]. Evidence from Norway spruce indicates climate-driven reductions in resistance under severe scenarios [6]. More broadly, climate change is amplifying extreme events [54,55], shifting host and pathogen phenology, disrupting hydraulic and carbon balance [56], reducing defensive compounds [57], and increasing pathogen fitness and transmission [58–60]. Storms interacting with decay pathogens further elevate windthrow risk. Collectively, warming, moisture extremes, and rising CO_2_ are reshaping host-pathogen synchrony and expanding infection windows, with major implications for forest health under climate change [61].

## Climate change introduces further complexity by perturbing forest tree immune layers at multiple scales

**Figure 2:**
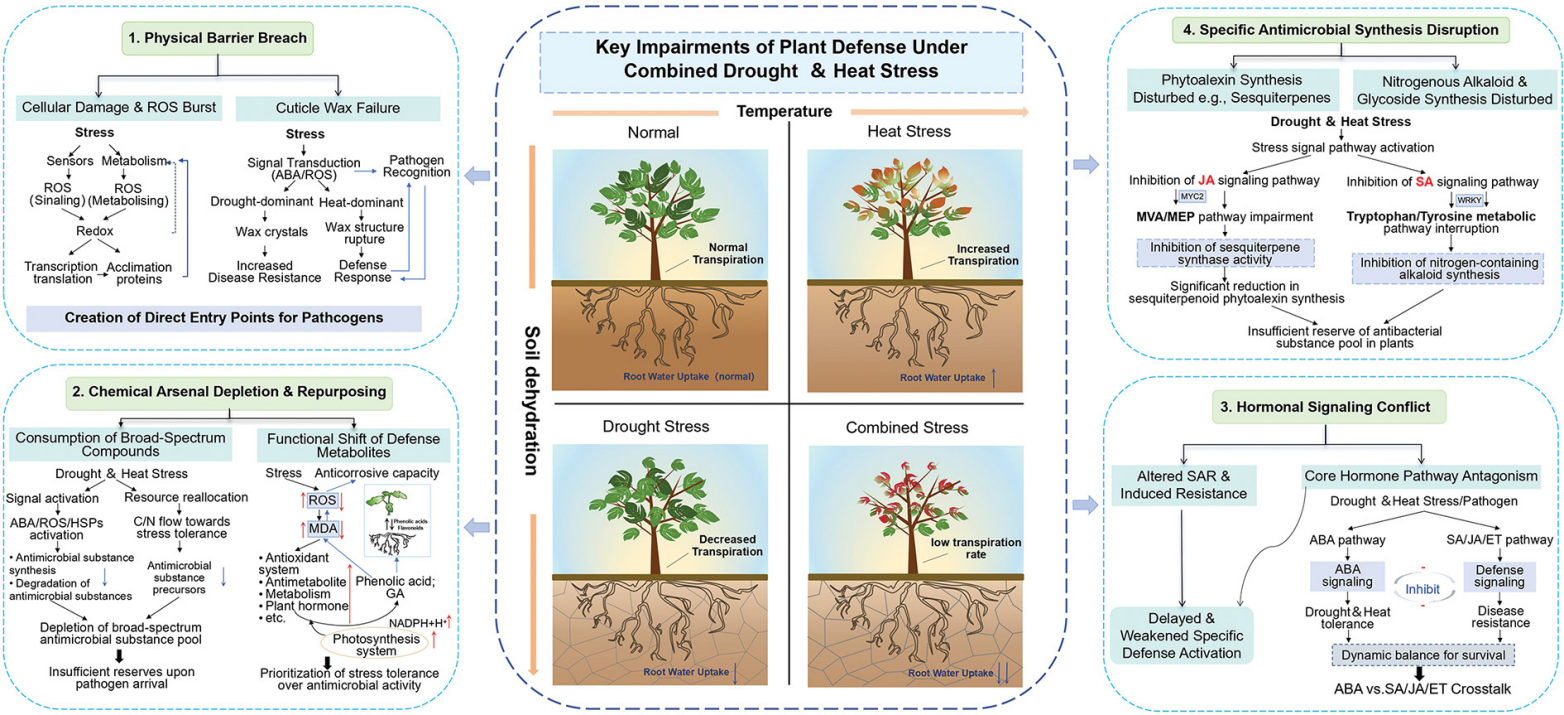
Plant defense systems under combined drought and heat stress Combined drought and heat stress weaken plant defense systems by impairing physical barriers, depleting antimicrobial compounds, disrupting hormonal signaling, and inhibiting the synthesis of phytoalexins and other defensive metabolites, ultimately reducing disease [7–11]. ROS (reactive oxygen species), ABA (abscisic acid), HSP (heat shock protein), GA (gibberellic acid), MDA (malondialdehyde), JA (jasmonic acid), SA (salicyclic acid), ET (ethylene), SAR (systemic acquired resistance), MYC2 & WRKY (transcription factors).

From a mechanistic perspective, climate-induced immune reconfiguration can be understood as a coordinated cascade linking stress perception, signal transduction, transcriptional regulation, and metabolic reprogramming (Figure 2). Drought, elevated temperature, and rising CO_2_ initially disturb cellular redox homeostasis, leading to the accumulation of ROS species such as H_2_O_2_ and O_2_^−^. Beyond their damaging effects, these molecules function as secondary messengers that activate calcium influx, mitogen-activated protein kinase signaling pathways, and redox-sensitive transcription factors, thereby initiating large-scale transcriptomic responses [26,27]. ROS signaling is closely integrated with phytohormonal networks, particularly ABA (abscisic acid), SA (salicyclic acid), JA (jasmonic acid), and ET (ethylene). Under drought and heat stress, ABA accumulation promotes stomatal closure and stress acclimation but also modifies ROS production and signaling, creating regulatory nodes that influence both abiotic stress tolerance and pathogen defense. Through extensive cross-talk, these hormonal pathways determine whether cellular resources are allocated toward growth, stress acclimation, or immune responses.

A major downstream consequence of this signaling network is the transcriptional reprogramming of secondary metabolism, especially the phenylpropanoid pathway. Transcriptomic studies in trees have shown that climate stress frequently alters expression of genes involved in phenylalanine metabolism, lignin biosynthesis, flavonoid production, and tannin accumulation [62,63]. These metabolites serve dual functions as antimicrobial compounds and antioxidants. Consequently, climate stress can alter not only the quantity but also the functional deployment of phenylpropanoid-derived metabolites. Under moderate stress, enhanced flavonoid and phenolic synthesis may strengthen antioxidant capacity and contribute to defense priming. However, prolonged drought or heat often redirects metabolic flux toward ROS detoxification, osmotic adjustment, and cell-wall stabilization, reducing the availability of defensive compounds specifically targeted against pathogens. This metabolic shift provides a biochemical explanation for the frequently observed decline in pathogen resistance under chronic climate stress.

Transcription factors act as critical integrators of these responses. Members of the WRKY, MYB, NAC, and HSF families coordinate hormone-responsive gene expression with phenylpropanoid biosynthesis, antioxidant metabolism, and stress adaptation. For example, HSF-mediated interactions with ABA signaling regulate heat-induced stress responses, whereas WRKY-associated networks influence JA- and SA-dependent defense pathways while simultaneously modulating secondary metabolism. Such regulatory modules illustrate how climate stress and immunity converge at the transcriptional level, producing coordinated changes in both gene expression and metabolite accumulation.

Empirical evidence indicates that drought and warming reconfigure tree defenses at transcriptomic, metabolic, and physiological levels. Reduced non-structural carbohydrate reserves can limit the production of terpenoids and other defensive metabolites, linking carbon availability directly to resistance. In Norway spruce, drought increased pathogen-related mortality while repressing defense-associated genes and downregulating pathways related to ABA, carotenoids, phenolics, and photosynthesis [63,64]. In *Pinus edulis*, drought impaired mono- and sesquiterpene defenses through hydraulic constraints on resin transport, demonstrating that resistance loss can result from disrupted defense deployment as well as altered biosynthesis [65,66]. Sustained heat similarly promotes transcriptomic reprogramming that prioritizes survival and repair over chemical defense [67], although responses may vary among genotypes and species [68].

Together, these studies suggest that climate stress does not simply weaken defense but reallocates resources from active resistance toward maintenance, repair, and stress tolerance [69]. Evidence from chestnut, where warming altered phenolic composition, antioxidant gene expression, and susceptibility, further supports the role of climate-driven metabolic reprogramming in shaping disease outcomes [70]. Tree immunity under climate change is therefore better viewed as a rewired system operating under shifting physiological priorities, where resistance reflects trade-offs among defense, growth, and stress adaptation rather than a simple reduction in defensive capacity.

## Epigenetic and transgenerational effects on tree responses to pathogens under climate change

In forest trees, where long generation times may constrain genetic adaptation to emerging disease pressures, epigenetic mechanisms could represent an additional layer of response. This possibility is particularly relevant for climate-sensitive disease systems such as ash dieback caused by *Hymenoscyphus fraxineus*. Since the 1990s, ash dieback has caused widespread decline of European ash (*Fraxinus excelsior*), with mortality often exceeding 60%–80% [71,72]. Disease severity is strongly influenced by climatic conditions: summer precipitation and humidity are associated with increased crown defoliation [73], whereas elevated summer and autumn temperatures can constrain fungal performance and reduce host damage and shoot mortality [74]. Future climate change is therefore expected to alter the distributions of both host and pathogen across Europe [75]. While these patterns are commonly interpreted through direct ecological effects on pathogen establishment and survival, they may also reflect climate-mediated changes in host regulatory processes. Environmental conditions that modify stress signaling, hormone balance, ROS homeostasis, and defense metabolism could influence epigenetic states that shape transcriptional responses to infection.

Consistent with this possibility, epigenomic variation associated with pathogen infection has been documented in European ash (*F. excelsior*) affected by ash dieback, where susceptible and tolerant genotypes differed at the whole-genome methylation level [76]. Such methylation differences may influence the expression of genes involved in defense signaling, phenylpropanoid metabolism, and stress responses, providing a potential mechanistic link between environmental conditions and disease outcomes. Similarly, pathogen infection in *Paulownia fortunei* has been associated with extensive changes in histone methylation and acetylation affecting genes linked to secondary metabolism, hormone signaling, and plant–pathogen interactions [77], while in poplar, infection by *Lonsdalea populi* induced global methylation changes correlated with resistance differences among genotypes [78]. Together, these studies suggest that epigenetic regulation may function as an interface through which climate-driven environmental signals are integrated with defense-associated transcriptomic and metabolic pathways. However, the available evidence remains largely correlative and does not yet establish whether epigenetic variation is causally involved in defense responses or instead reflects downstream consequences of infection, genotype effects, or other confounding factors. Demonstrating causality remains a major challenge, particularly in trees where functional epigenomic manipulation is still limited. Furthermore, current evidence for transgenerational epigenetic inheritance in trees remains suggestive rather than definitive, including maternal environmental effects in which offspring from pathogen-exposed mother trees sometimes exhibit enhanced tolerance [79,80]. Although such observations are consistent with epigenetic priming, direct mechanistic evidence remains scarce. Future progress will depend on experimental approaches that move beyond association studies, including longitudinal epigenome profiling, common-garden and reciprocal inoculation experiments, and emerging functional epigenetic tools to test causal links between chromatin states and disease resistance.

## Pathogen adaptation, distribution shifts, range expansion and invasion biology, and evolutionary responses

Novel climate regimes are reshaping the distribution and evolution of tree pathogens, with major consequences for forest ecosystems. Warming and altered precipitation relax climatic barriers, enabling poleward and upslope range expansions, longer transmission windows, and colonization of previously unsuitable habitats [12,81,82]. These processes are exemplified by *Phytophthora ramorum*, the causal agent of sudden oak death, which is established in North America and Europe, infecting oaks (*Quercus* spp.), tanoak (*Notholithocarpus densiflorus*), and numerous other hosts. Extensive mortality in U.S. oak forests and widespread ramorum blight in forests, nurseries, and gardens illustrate how climate-driven environmental change can enhance pathogen persistence and spread [83].

Evidence from *Phytophthora* spp. demonstrates strong climatic filtering across latitudinal and altitudinal gradients, with thermophilic, drought-tolerant taxa dominating warmer regions, while cold-adapted species persist at higher elevations and latitudes [84–86]. However, climate alone does not determine establishment; dispersal limitations, host availability, and local biotic interactions also play important roles [87]. Climate change further interacts with anthropogenic drivers, particularly global trade, which has facilitated the spread of invasive pathogens through the movement of live plants and nursery stock [88–90].

Pathogen populations are also evolving under these pressures, with selection favoring thermotolerant and drought-adapted traits [84,91,92]. In *Phytophthora*, thick-walled oospores and higher thermal growth optima may enhance persistence under heat and water stress [82,84,86,93]. Nevertheless, adaptive responses are constrained by trade-offs and vary among species. Collectively, forest disease risk under climate change emerges from interacting climatic shifts, pathogen adaptation, biological invasions, and community-level processes, rather than direct climatic control alone [87].

## Tree microbiomes: from disease modulation to microbiome

**Figure 3:**
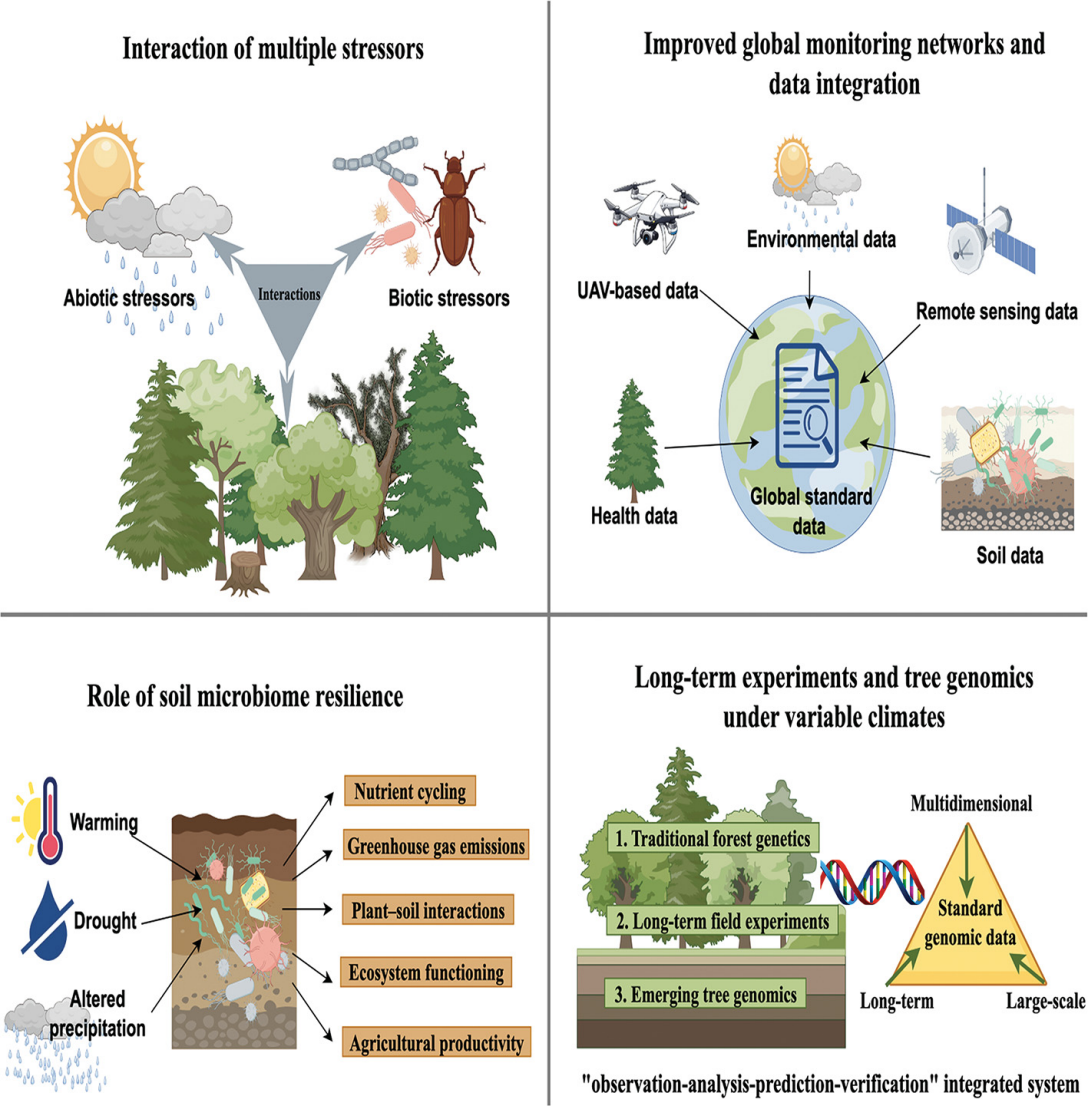
Future research directions An illustration of potential knowledge gaps and future research directions that integrates multidimensional datasets.

In forest ecosystems, the rhizosphere is a critical interface where roots, soil microbes, and pathogens interact to influence tree health (Figure 3) [94,95]. Rhizosphere and endophytic microbiota can suppress fungal pathogens through competition, antibiosis, and induced systemic resistance, while also enhancing nutrient acquisition and host growth [96,97]. These interactions are increasingly shaped by climate change, as elevated CO_2_, warming, drought, and flooding alter plant physiology, root exudation, and microbial community composition, often with contrasting effects across fungal and bacterial taxa [98–104]. Notably, responses are often highly variable, with outcomes dependent on host genotype, soil conditions, microbial community context, and interacting environmental stressors. Elevated CO_2_ may promote plant growth-promoting fungi, whereas bacterial responses are less consistent; warming, drought, and flooding can disrupt mycorrhizal and nitrogen-fixing communities while sometimes favoring pathogens [98–103].

This context dependency remains a major challenge for reproducibility. Although controlled studies often demonstrate strong biocontrol potential, field performance is frequently inconsistent because microbiome functions emerge from complex, co-evolved, and dynamic consortia that are difficult to replicate experimentally (Figure 3) [94]. High-dimensional microbiome datasets, spatiotemporal heterogeneity, and multifactor climate effects further complicate mechanistic inference and cross-site reproducibility. These limitations are particularly relevant for microbiome engineering, which has emerged as a sustainable alternative to chemical control by promoting disease-suppressive communities and enhancing resilience under combined abiotic and biotic stress [105,106]. However, establishing stable introduced consortia and achieving predictable outcomes across heterogeneous forest environments remain difficult under variable field conditions [107,108]. Thus, while microbiome-based strategies hold considerable promise for strengthening tree health and disease resilience [109], their practical deployment will depend on improving reproducibility and resolving variability across natural systems.

## From tree mortality to ecosystem feedback: composition, carbon, and biodiversity

Climate change is increasingly recognized as an indirect but powerful driver of forest transformation through its interactions with plant pathogens. By weakening host resistance while expanding pathogen distributions and activity, climate stress can increase tree mortality and accelerate shifts in forest composition and successional trajectories [110–112]. These disease impacts are not merely episodic disturbances; they can act as mechanisms of ecological reorganization through the selective loss of dominant species. For example, mortality of *Pinus thunbergii* and *P. massoniana* caused by the pine wilt nematode (*Bursaphelenchus xylophilus*) has driven succession toward broadleaved communities dominated by *Liquidambar formosana* and *Quercus acutissima* in subtropical China [111,113]. Furthermore, climate change is expected to intensify pine wilt disease by increasing host drought stress and nematode reproduction [114,115] Models predict expansion from the Mediterranean into northern Europe and poleward shifts in North America, increasing future risk at higher latitudes [116,117]. Additionally, mortality of tanoak (*N. densiflorus*) caused by *P. ramorum* has altered stand structure and reduced diversity in California forests [110]. Such transitions may maintain forest cover but fundamentally alter ecosystem composition, function, and resilience, particularly where feedbacks or regeneration constraints limit recovery of affected species.

Disease-mediated mortality also has important implications for the global carbon cycle. Large-scale tree death releases stored carbon through decomposition and disturbance while reducing future carbon sequestration capacity [118]. In western North America, bark beetle outbreaks have caused extensive pine mortality, reducing forest carbon uptake and increasing emissions [119]. Between 1984 and 2010, fires and beetle outbreaks in the western U.S.A. killed trees containing an estimated 5–11 and 2–24 Tg C yr^−1^, respectively, together representing approximately 9% of total tree carbon stocks [120]. Climate extremes can further amplify these effects. The 2011–2015 California drought killed more than 140 million trees and contributed to a regional shift toward a net carbon source, with approximately 600 Tg CO_2_ emitted between 2001 and 2015 [121]. Projections of continued ecosystem carbon decline suggest that mortality-driven carbon losses may create reinforcing climate–forest feedbacks that weaken the role of forests as long-term carbon sinks [121]. Beyond carbon dynamics, climate-driven disease outbreaks can alter biodiversity, forest structure, and hydrological processes. Increased disease incidence and mortality contribute to changes in species composition and forest cover, with consequences for ecosystem functioning [122,123]. Canopy loss and compositional change affect interception, transpiration, evaporation, and water yield [124–126]. In the Oregon Coast Range, warmer winters are projected to increase the severity of Swiss needle cast, and where infection exceeds 10%, reduced canopy interception and transpiration may increase streamflow and alter seasonal water dynamics [127,128]. However, these responses are context-dependent. Although canopy decline may temporarily increase water yield, longer-term changes in flow timing, evapotranspiration, and species composition may reduce watershed stability and resilience.

Collectively, these examples demonstrate that climate-driven disease dynamics extend far beyond individual host–pathogen interactions. By influencing succession, carbon storage, biodiversity, and hydrology, pathogen-induced mortality represents a key pathway through which climate change reshapes forest ecosystems, with effects that may persist long after the initial outbreak [110–113,118–128].

## Integrated strategies for managing tree diseases in a changing climate

Breeding and assisted migration are increasingly important for enhancing tree resilience to climate change and emerging diseases, but their effectiveness depends on integration with ecosystem-level management. Modern breeding is moving beyond phenotype-based selection toward climate-informed screening, genomic prediction, and analyses of adaptive regulatory pathways [129–132]. Epigenetic mechanisms, including DNA methylation and small RNAs, may provide additional opportunities for resilience enhancement, although their practical application remains largely experimental [133–137]. Assisted migration is likewise becoming more targeted through climate projections and genome–environment analyses, but ecological risks and uncertainties remain significant.

Disease management increasingly emphasizes diversity-based approaches. Greater species and phylogenetic diversity can reduce pathogen transmission and enhance resilience through complementary defense traits [42,138–142]. Practices such as thinning, retention of native species, and targeted sanitation can further strengthen adaptive capacity, particularly when tailored to different stages of pathogen invasion [138]. However, climate change and global trade are generating increasingly novel disease dynamics, highlighting the need for effective surveillance and early-warning systems.

Predictive modelling and monitoring are becoming essential components of forest health management. UAV-based remote sensing, deep learning, and satellite imagery have improved detection of tree decline and mortality [143–146], while mechanistic models provide insights into physiological thresholds and future climate risks [147,148]. Integrating these tools within adaptive management frameworks will be critical for anticipating emerging threats and improving forest resilience under rapid environmental change.

## Knowledge gaps in tree–pathogen interactions under climate change

Earth system models frequently treat abiotic and biotic drivers as separate processes, which can reduce ecological realism and limit predictive accuracy, particularly when estimating risk thresholds (Figure 3). This separation is problematic because ecological responses often emerge from interactions among multiple stressors rather than from isolated drivers. For example, although UAV-based monitoring has shown strong potential for detecting visible disease symptoms, its performance often declines when stressors such as drought and pathogen pressure co-occur, revealing current limitations in capturing compound disturbance dynamics [144,149]. Addressing this challenge requires modelling frameworks that explicitly integrate environmental and biological drivers, supported by multi-temporal, high-resolution datasets that can improve the robustness of deep-learning applications and large-scale monitoring systems [146,147,150]. At the same time, progress depends not only on greater data volume but also on conceptual advances that move beyond correlative approaches toward more mechanistic, process-informed models. Integrating forest genetics, long-term ecological experiments, and tree genomics is increasingly recognized as essential for predicting climate adaptation, yet substantial knowledge gaps remain [151,152]. A major limitation is that many genome-wide studies still rely on short-term or single-time-point phenotypic observations, restricting understanding of long-term plasticity, adaptive capacity, and the architecture of complex traits under interacting stresses [153,154]. This shortcoming is compounded by the lack of standardized multidimensional datasets, which constrains synthesis across scales and disciplines (Figure 3). Critical priorities therefore include long-term genotype-environment experiments, stronger cross-scale and interdisciplinary collaboration, development of gene-informed predictive models, and open research infrastructures to improve resilience forecasting. However, realizing these priorities will require overcoming persistent barriers related to data interoperability, experimental comparability, and integration between molecular and ecosystem-scale research.

Similar challenges arise in understanding belowground processes. Soil microbial communities (Figures 2 and 3), which underpin nutrient cycling, greenhouse gas fluxes, and broader ecosystem functioning, are extraordinarily diverse and functionally significant [155–157]. Although they are often assumed to be resilient because of rapid turnover and functional redundancy [158], empirical evidence suggests their responses to warming, drought, and altered precipitation are highly context-dependent and often nonlinear [94,159,160]. Moreover, resilience varies according to stress type, intensity, and duration [161,162], complicating generalized predictions. This variability highlights a critical research gap: identifying region-specific drivers and thresholds that govern microbial responses, which remains essential for ecosystem management and forecasting [61,81,155]. A critical discussion must therefore question simplified assumptions of microbial resilience and recognize that redundancy does not necessarily ensure stability under chronic or interacting disturbances. Despite rapid advances in remote sensing, globally coordinated monitoring systems and standardized datasets remain limited, reducing model transferability and contributing to spatial-temporal mismatches between UAV and satellite observations [144,163]. These limitations expose a broader challenge in scaling ecological observations from individual trees to regional and global assessments. Future research should prioritize standardized monitoring frameworks that link fine-scale UAV observations with continuous satellite-based surveillance [149], alongside data-fusion approaches that integrate climate, soil, physiological, and biotic disturbance variables to improve large-scale forest health predictions (Figure 3) [148]. Critically, however, technological innovation alone is unlikely to resolve these issues unless accompanied by coordinated international data-sharing efforts, harmonized protocols, and modelling frameworks capable of representing cross-scale ecological complexity. Taken together, advancing resilience prediction will depend as much on improving integration across disciplines, data streams, and scales as on developing new sensing or analytical technologies.

## Future research priorities: next-generation strategies for climate-resilient forest tree disease management

Addressing forest tree diseases under accelerating climate change will require integrating conceptual and technological breakthroughs from multiple disciplines, including medicine, computational biology, and synthetic biology. Translational innovation adapting biomedical advances to long-lived forest species offers a particularly promising direction. Precision genome editing technologies such as CRISPR-based systems are expected to play a central role in fine-tuning endogenous immunity pathways [164]. Rather than introducing foreign genes, targeted editing of susceptibility genes, promoter regions, or immune receptor repertoires could enhance quantitative resistance while preserving local adaptation. Emerging tools such as base editing and prime editing further enable precise nucleotide modifications without double-strand breaks, potentially reducing unintended genomic effects in long-lived tree genomes. In parallel, RNA-based technologies inspired by vaccine platforms developed during the COVID-19 pandemic offer novel opportunities for transient immune activation. Exogenous application of double-stranded RNA [165,166], small interfering RNA, or synthetic mRNA constructs could temporarily induce defense pathways or silence pathogen virulence genes without permanently altering tree genomes. Such spray-induced gene silencing approaches may provide flexible, seasonally deployable protection strategies, particularly valuable in high-value forestry or conservation contexts.

Beyond direct genetic intervention, predictive and systems-level approaches will increasingly define the next generation of resistance breeding. Artificial intelligence (AI)-assisted breeding frameworks that integrate pan-genomic variation, epigenomic regulation, climate projections, and pathogen evolutionary modelling could shift forestry from retrospective selection toward forecast-driven design. Unlike conventional genomic selection, these integrative platforms would model polygenic resistance architectures under future climate–pathogen scenarios, identifying genotypes optimized for durability, plasticity, and adaptive capacity. Coupling machine learning with long-term field phenotyping networks and remote sensing data (e.g., drone and satellite-based stress detection) could further refine early disease prediction and resistance assessment. Ultimately, durable disease management in forest trees will depend on integrating molecular innovation with ecological realism. Long generation times, complex genomes, and heterogeneous environments demand approaches that combine precision genetics, transient bio-based interventions, and predictive modelling. A coordinated effort bridging molecular biology, evolutionary theory, computational science, multi-omics, and forest management will be essential to build resilient forests capable of withstanding rapidly evolving pathogen pressures in a changing world.

## Summary

- Climate change increases forest disease risks by altering environmental conditions, promoting pathogen spread and outbreaks, and changing tree physiological and molecular stress responses that affect resistance.
- Epigenetic mechanisms, including DNA methylation, histone modifications, and small RNAs, are increasingly recognized as important contributors to tree responses to pathogens under climate change.
- Climate-induced changes not only intensify plant and tree disease impacts but also challenge existing disease management strategies, underscoring the need for adaptive and climate-resilient approaches in plant and forest health management.
- Understanding forest tree responses to pathogens under climate change requires multidisciplinary approaches that integrate molecular biology, genomics, big data analytics, remote sensing, ecology, and climate modeling.
- Innovative directions and transformative approaches for durable forest health would require the deployment of precision genome editing technologies such as CRISPR, mRNA-based technologies, and AI-assisted breeding frameworks.

## Open Access

Open access for this article is provided via our Subscribe to Open programme, made possible through the support of our subscribing institutions. Learn more here: https://portlandpress.com/pages/open_access_options_and_prices#conditional.

## Abbreviations

ABA: abscisic acid
AI: artificial intelligence
CRISPR: Clustered Regularly Interspaced Short Palindromic Repeats
ET: ethylene
ETI: Effector triggered immunity
GA: gibberellic acid
JA: jasmonic acid
PTI: Pattern triggered immunity
ROS: reactive oxygen species
SA: salicyclic acid
UAV: Unmanned Aerial Vehicle
